# Purification and Characterization of Gum-Derived Polysaccharides of *Moringa oleifera* and *Azadirachta indica* and Their Applications as Plant Stimulants and Bio-Pesticidal Agents

**DOI:** 10.3390/molecules27123720

**Published:** 2022-06-09

**Authors:** Nagarajan Shobana, Pandurangan Prakash, Antony V. Samrot, P. J. Jane Cypriyana, Purohit Kajal, Mahendran Sathiyasree, Subramanian Saigeetha, T. Stalin Dhas, D. Alex Anand, Gokul Shankar Sabesan, Bala Sundaram Muthuvenkatachalam, Basanta Kumar Mohanty, Sridevi Visvanathan

**Affiliations:** 1 School of Bio and Chemical Engineering, Sathyabama Institute of Science and Technology, Sholinganallur, Rajiv Gandhi Salai, Chennai 600119, India; shobanan1993@gmail.com (N.S.); janea521@gmail.com (P.J.J.C.); kajalpurohit13@gmail.com (P.K.); sathiyasree02@gmail.com (M.S.); danielalexanand@gmail.com (D.A.A.); 2 School of Bioscience, Faculty of Medicine, Bioscience and Nursing, MAHSA University, Jalan SP2, Bandar Saujana Putra, Jenjarom 42610, Malaysia; 3 Department of Biotechnology, School of Biosciences and Technology, Vellore Institute of Technology, Vellore 632014, India; rajivarsha2000@gmail.com; 4 Centre for Ocean Research, MoES—Earth Science & Technology Cell, Col. Dr. Jeppiaar Research Park, Sathyabama Institute of Science and Technology, Chennai 600119, India; stalindhas.cor@sathyabama.ac.in; 5 Faculty of Medicine, Manipal University College Malaysia, Jalan Batu Hampar, Bukit Baru, Melaka 75150, Malaysia; gokkavi@gmail.com (G.S.S.); drbasantmohanty@gmail.com (B.K.M.); 6 Faculty of Medicine, AIMST University, Batu 3 1/2, Jalan, Bukit Air Nasi, Bedong 08100, Malaysia; balabcaimst@gmail.com (B.S.M.); sridevimma@gmail.com (S.V.)

**Keywords:** plant gums, seed germination, soil efficiency, bio-pesticidal activity

## Abstract

Plant gums are bio-organic substances that are derived from the barks of trees. They are biodegradable and non-adverse complex polysaccharides that have been gaining usage in recent years due to a number of advantages they contribute to various applications. In this study, gum was collected from *Moringa oleifera* and *Azadirachta indica* trees, then dried and powdered. Characterizations of gum polysaccharides were performed using TLC, GC-MS, NMR, etc., and sugar molecules such as glucose and xylose were found to be present. Effects of the gums on *Abelmoschus esculentus* growth were observed through root growth, shoot growth, and biomass content. The exposure of the seeds to the plant gums led to bio stimulation in the growth of the plants. Poor quality soil was exposed to the gum polysaccharide, where the polysaccharide was found to improve soil quality, which was observed through soil analysis and SEM analysis of soil porosity and structure. Furthermore, the plant gums were also found to have bio-pesticidal activity against mealybugs, which showed certain interstitial damage evident through histopathological analysis.

## 1. Introduction

The use of chemicals and synthetic fertilizers has been growing consistently over the recent decades, causing adverse effects to the non-target environment, and posing a threat to the ecosystem [1]. However, many sustainable alternatives provided by nature have been used due to their non-toxic properties. Examples of such products are plant gums. They are bioorganic substances that are secreted from the barks of trees in response to any stress or damage/wound caused by either biotic or abiotic sources [2]. Plant gums are complex carbohydrate polymers produced predominantly by herbaceous and woody plants belonging to the Fabaceae, Combretaceae and Sterculiaceae families [3]. Gums are mostly made up of polysaccharides, which belong to the group of complex carbohydrates [4] and are known to be hydrophilic in nature [5]. They have been widely used in recent decades due to their unique properties and the functionalities in various fields that they contribute through numerous applications [6]. These gum exudates are insoluble in organic solvents. However, they have an ability to produce viscous gels when they are added to an aqueous system. They also create intermolecular bonds with two or more polymers, resulting in the formation of a three-dimensional network that traps water molecules. They are held together by hydrogen bonding or ionic interactions and thus, a gel is formed [7,8]. They also possess high tensile strength, are innocuous in nature, and thus can be useful in improving soil efficiency [9]. Gum polysaccharides are highly biocompatible and biodegradable in nature because they truly represent renewable sources and are composed of repeated monosaccharide units. Therefore, they have no adverse impact on human health and the environment [10].

*Azadirachta indica* (AI), commonly known as neem is a tropical evergreen tree native to the Indian sub-continent. Various parts of the trees have been exploited in a number of applications for the past 4000 years due to their unique properties [11]. The leaves, twigs, and seeds of neem tree have many medicinal uses. Neem twigs were used for tooth cleaning to combat various skin ailments, neem leaf juice was consumed as a tonic, and neem leaves were placed in beds, books, grain bins, cupboards, and closets to ward off pesky bugs [12,13]. Neem gum, which can be obtained as an exudate from the bark of the *Azadirachta indica* tree, is a complex hetero-polysaccharide that is clear and brown in color [14]. It belongs to the family of galactan gums and has been investigated for its antioxidant [15], anticancer [16] and anti-inflammatory properties [17]. Among its numerous distinguishing characteristics, neem gum contains a significant amount of D-glucosamine and proteins, making it an ideal experimental material for studying the biological activities of proteins in exudate gums [18]. It was used for aqueous film coating in the formulation of tablets [19]. It can also be used for the preparation of diabetic-friendly meals [20]. *Moringa oleifera* (MO), also known as the “Miracle Tree”, is a highly valued plant that is predominantly cultivated in the tropics and subtropics. Leaves, flowers, and immature pods of *Moringa oleifera* are suitable for consumption due to their high content of amino acids, vitamins, minerals, proteins, fiber, and lipids [21] and as food additives against food-borne pathogens such as *Escherichia coli*, *Pseudomonas aeruginosa*, *Staphylococcus aureus*, *Salmonella typhimurium* and *Enterobacter aerogenes* [22]. Anti-asthmatic, anticancer, analgesic, antidiabetic, antipyretic, wound healing, antispasmodic, and antiulcer properties have also been observed [23]. White gum secreted from the stem of *Moringa oleifera*, a member of the Moringaceae family, turns to a brownish color on exposure to the sun and the environment [24]. It is partially soluble in water and forms a highly viscous solution in contact with water [25] and is also used as a binder in tablet formulations, playing an important role in drug release retardation [26].

Infestations by *Phenacoccus solenopsis* (cotton mealy bugs) in greenhouses can be observed all year and are most commonly visible on shoot tips and the undersides of leaves, covering them in vast amounts of white waxy material [27]. These are quite aggressive in nature and feed by inserting their mouth parts into the plant phloem and sucking the sap [28]. Due to its high reproductive capacity, *Phenacoccus solenopsis* has the potential to harm or destroy native plant species. Pesticides can be used to get rid of them; however, the presence of the white waxy coating makes eradication difficult. Limited studies are available on the applications of these two gums as plant growth promoters and bio-pesticides. Their polysaccharides are similar to the polysaccharides generated by soil microbes. They are superabsorbent and aid in the modification of water retention capacity and the improvement of soil structure and physical qualities. These gums have been used in the past to remove oils and heavy metals from soil [29,30,31] and biocides as well [32,33,34]. Plant gums can be used to stimulate plant growth. *Acacia senegal* and its nitrogenous derivatives, such as arabic acid, ammonium arabate, arabamide, fraction of high protein, and fraction of low protein, were utilized to promote growth in Winka seeds. The number of leaves, height, and dry weight of plants treated with the gum and its compounds differed significantly [35]. Therefore, in this study, the above-mentioned plant gums were investigated for improvement of plant growth and soil efficiency and to investigate their pesticidal activity.

## 2. Results

### 2.1. Characterization of Crude Gum 

The phytochemical analysis of the MO gum revealed the presence of phytochemical compounds such as alkaloids, saponins, reducing sugars, phytosterols, tannins and glycosides. Likewise, AI gum contained alkaloids, flavonoids, saponins, reducing sugars, tannins and glycosides (Supplementary Table S1). Mucilage from *Opuntia cochenillifera* (L.) has been reported to possess alkaloids and terpenes/steroids by the Dragendorff, Mayer, and Wagner tests [36]. Water extract of *Acacia tortilis* gum contains flavonoids, saponins, steroid, quinone, and tannin as confirmed by thin layer chromatography [37]. The aqueous solution of gum was subjected to UV-visible spectroscopy analysis in the range of 200–800 nm. The UV-Vis graph of *Moringa oleifera* gum displayed peaks around 200–240 nm and 279 nm (Supplementary Figure S3a). Similarly, AI gum also showed peaks at around 200–220 nm and at 258 nm (Supplementary Figure S3b). Supplementary Figure S4a,b shows FTIR spectrum of gum of *Moringa oleifera* and *Azadirachta indica*, respectively. The characteristic peak at 3673 cm^−1^ indicates OH stretching, which is the characteristic peak for the glycosidic bonds present in the polysaccharides, and a broad spectrum at 3279 cm^−1^ was displayed in *Moringa oleifera* gum, indicating the presence of a carboxylic acid group. The peaks at 2930 and 2933 cm^−1^ for MO and AI gum were attributed to the C-H stretching vibration in alkanes [38], and the peaks found between 1300–1450 cm^−1^ indicated the presence of –CH_3_ and –CH_2_ bending [39]. Peaks at 1285 and 1234 cm^−1^ specified the presence of C-N stretching in amine groups. Peaks in the range of 1610 to 1600 cm^−1^ corresponded to C=C stretching in α, β-unsaturated ketones. Both gums showed bands around 1000–1200 cm^−1^ that revealed the fingerprints of carbohydrates [40].

TLC was performed for MO and AI gum using the solvent systems ethanol:water (2:1) and ethanol:chloroform (2:1), respectively. R_f_—0.47, 0.52, 0.82 were obtained for *Moringa oleifera* gum (Supplementary Figure S5a) where R_f_—0.52, 0.76, 0.82 were found in *Azadirachta indica* gum (Supplementary Figure S6a). Rao and Beri [41] employed paper chromatography for identification of monosaccharides. Using TLC bioautography for antioxidant activity, it was revealed that gum has antioxidant property (Supplementary Figures S5b and S6b), also. As shown in Supplementary Figure S7a,b, the maximum absorbance in the phenol sulfuric acid spectrum was found between 430 and 450 nm, indicating the presence of monosaccharides in the MO gum [42]. The moisture content in MO gum and AI gum was 55 ± 0.32% and 4 ± 0.29%, respectively, while the ash content was 2.147 ± 0.07% and 2.23 ± 0.03%, respectively (results not shown in any table). An important factor in determining the structure and properties of polysaccharides is their moisture and ash content, which is usually composed of carbonates, phosphates, silicates, and silica [43]. Gum of *Acacia Senegal* (gum Arabic), of the Central Rift Valley of Ethiopia, has been reported to possess 15% moisture and 3.56% ash content [44]. In another report, the gum of *Acacia Senegal* was found to contain 12.02 ± 0.02% moisture and 2.45 ± 0.01% ash content, while xanthan gum contains 10.64 ± 0.55% moisture and 7.60 ± 0.05% ash content [45].

### 2.2. Characterization of Purified Gum Polysaccharide

**Figure 1:**
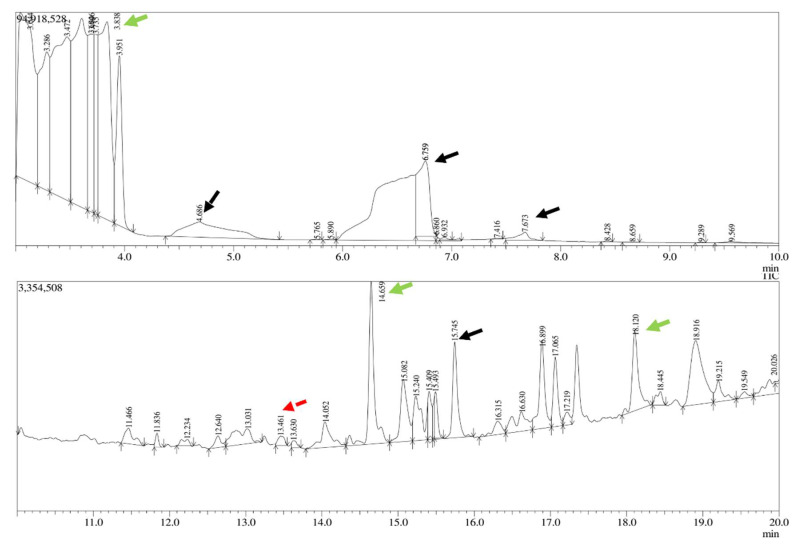
GC-MS analysis of MO gum polysaccharide. Peaks at 3.838, 14.656, 18.120 (green arrow)—glucose, peak at 4.686 (black dotted arrow)—D allose, peaks at 6.759, 7.673, 15.745 (black arrow)—derivatives of galactose, peak at 13.461 (red dotted arrow)—derivative of xylose.

**Figure 2:**
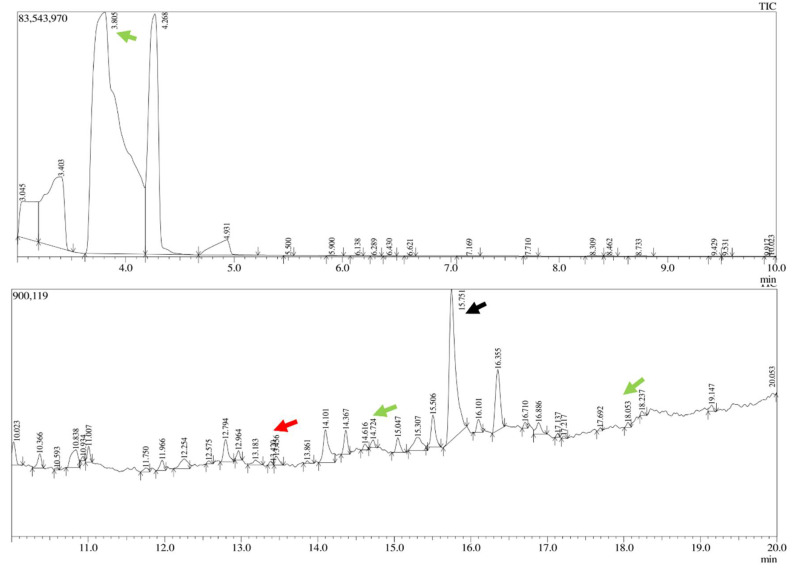
GC-MS analysis of AI. Peak at 3.805, 14.616, 18.053 (green arrow)—glucose, peak at 15.751 (black arrow)—derivatives of galactose, peak at 13.430 (red arrow)—derivative of xylose.

The gum polysaccharide (from MO and AI) was purified, and the absorbance maxima were detected in the range between 200 to 280 nm and at 410 nm for MO and AI gum polysaccharide, respectively (Supplementary Figure S8a,b). FT-IR analysis of MO and AI gum polysaccharide showed broad peaks in the range 3200 to 3400 cm^−1^, corresponding to O-H stretching of alcohol groups in the gum polysaccharide (Supplementary Figure S9a,b). The presence of peaks at 2361 and 2362 cm^−1^ indicated O=C=O stretching vibration. Alkene groups could be confirmed by peaks present at 1644 and 1637 cm^−1^. Peaks at 1516 cm^−1^ and 1140 cm^−1^ were attributed to C=C aromatic and aliphatic ether groups. GC-MS analysis of MO gum polysaccharide revealed the presence of various sugars and derivatives of sugar molecules such as glucose, D allose, galactose and xylose (Figure 1). Glucose, galactose and xylose were detected in the AI gum polysaccharide (Figure 2). Similar results have been reported by Samrot et al. [46].

**Figure 3:**
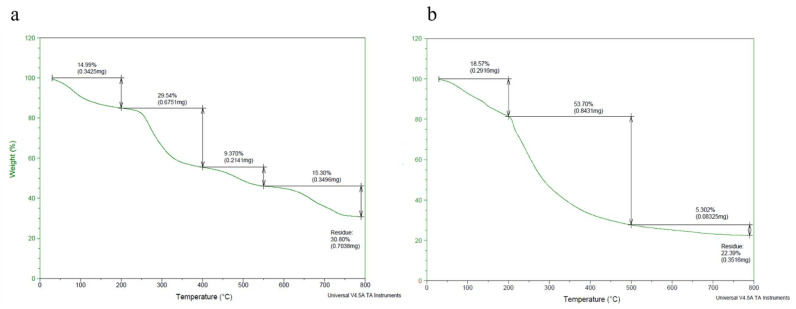
Thermogravimetric analysis of (**a**) MO gum, (**b**) AI gum.

**Figure 4:**
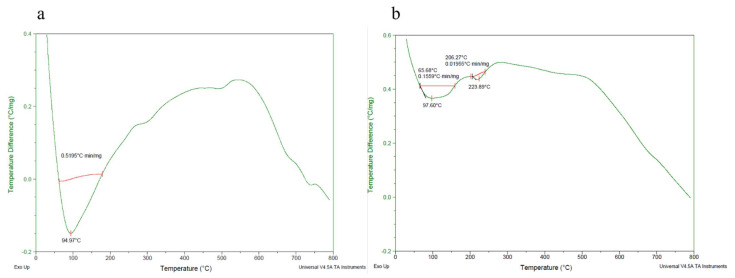
Differential thermal analysis of (**a**) MO gum, (**b**) AI gum.

**Figure 5:**
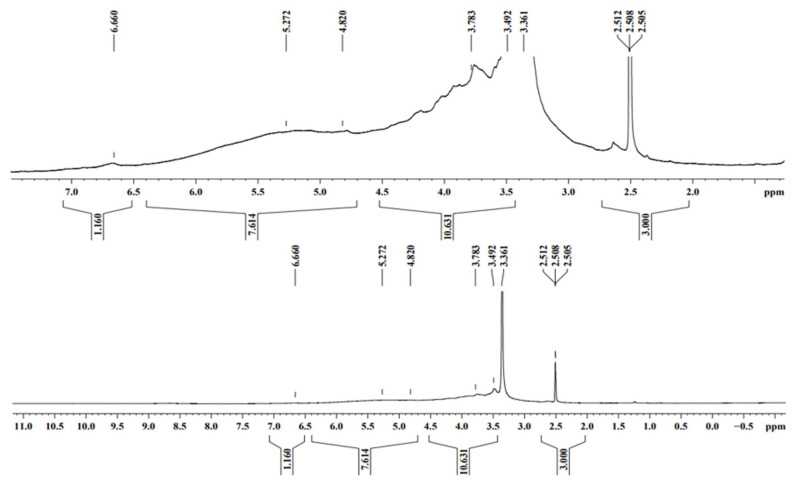
^1^H NMR spectra of MO gum.

**Figure 6:**
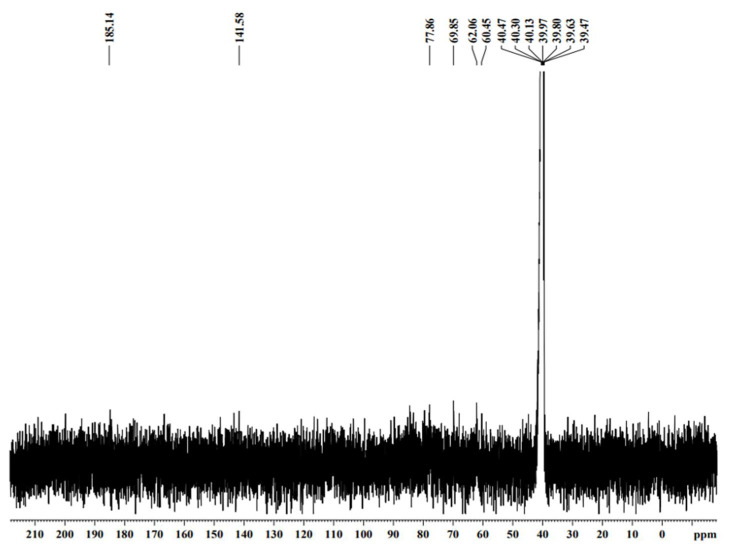
^13^C NMR spectra of MO gum.

**Figure 7:**
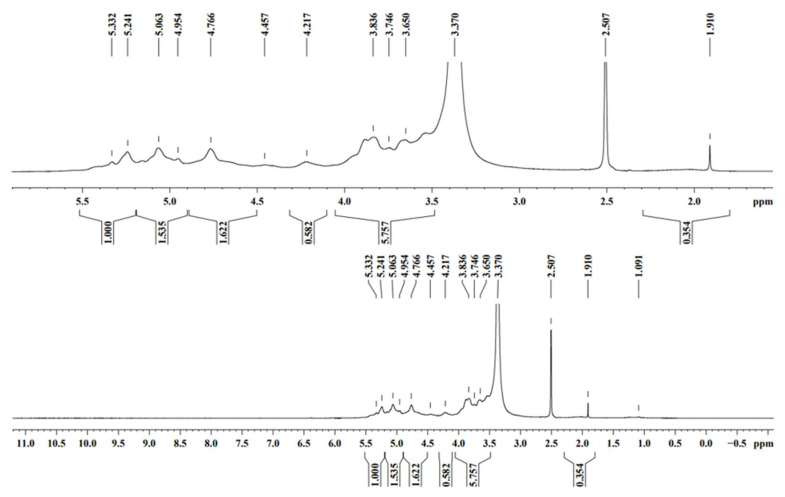
^1^H NMR spectra of AI gum.

**Figure 8:**
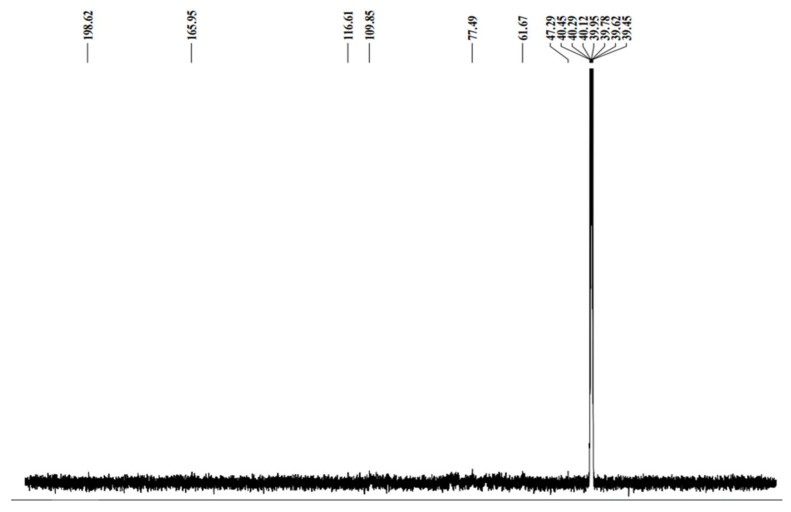
^13^C NMR spectra of AI gum.

The thermogravimetry of the MO and AI gums showed a weight loss from 100 °C and there was huge loss in gravimetry after crossing from 200 to 485 °C (Figure 3a,b). DTA thermograms of MO gum showed a major intense peak indicating an endothermic transition at around 100 °C followed by an exothermic transition at around 500 to 600 °C (Figure 4a). AI gum showed weaker endothermic transitions at 100 to 300 °C and exothermic transition at 500 to 600 °C (Figure 4b). Different gum polysaccharides have different structural and functional compositions, which cause different degradation routes [47,48]. The ^1^H NMR spectrum of MO gum polysaccharide revealed signals corresponding to the presence of galactose and xylose. The peak at 4.820 ppm revealed the presence of H-1 of β-glucose. The peak at the ppm of 5.27 and 3.782 shows the presence of the H-1 and H-2 of α-galactose. The peak at 3.361 ppm depicts the presence of β-xylose (Figure 5). ^13^C NMR spectra of MO gum revealed peaks at 77.8 ppm and 69.85 ppm corresponding to the presence of derivatives of β-glucose and α-D-galactose respectively (Figure 6). Similarly for AI gum polysaccharide, peaks at 4.766 ppm and 3.37 ppm revealed the presence of H-1 and H-2 of β-glucose sugar molecules. Signals corresponding to 5.332 and 3.650 ppm confirm the presence of H-1 and H-2 of α-glucose sugar molecules. The peak at 3.836 ppm showed the presence of H-1 of α-galactose (Figure 7). Derivatives of glucose at H-3 was revealed by the peak at 3.746 ppm. The derivatives of glucose sugar molecules were confirmed by the peak of ^13^C at 77.49 ppm. Sharp peak at 61.67 ppm revealed the presence of derivatives of β-D-Galactose (Figure 8). Similar results have been reported earlier [49,50,51].

### 2.3. In-Vitro Study on Plant Growth

**Figure 9:**
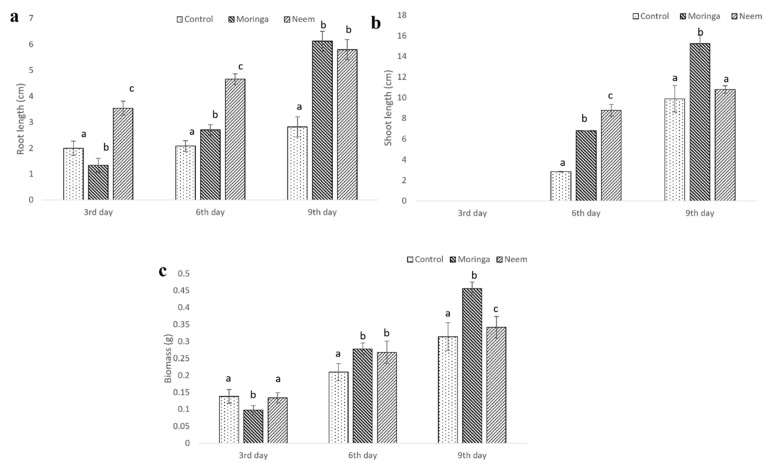
In vitro phyto stimulation of *A. esculentus* exposed to MO and AI gum polysaccharide: (**a**) root growth, (**b**) shoot growth, (**c**) biomass. All tests are compared with control. Different letters on the bar graph denote significant difference at *p* < 0.05.

Evaluation of the gum for their plant growth-inducing activity showed that gum polysaccharide-enriched seeds had a higher germination rate than control seeds for MO plant gum (Table 1). Both gums stimulated the root and shoot growth on the 6th and 9th day of the study. The rate of growth was found to increase with increased time of exposure to gum (Figure 9).

### 2.4. In-Vivo Study on Plant Growth

**Figure 10:**
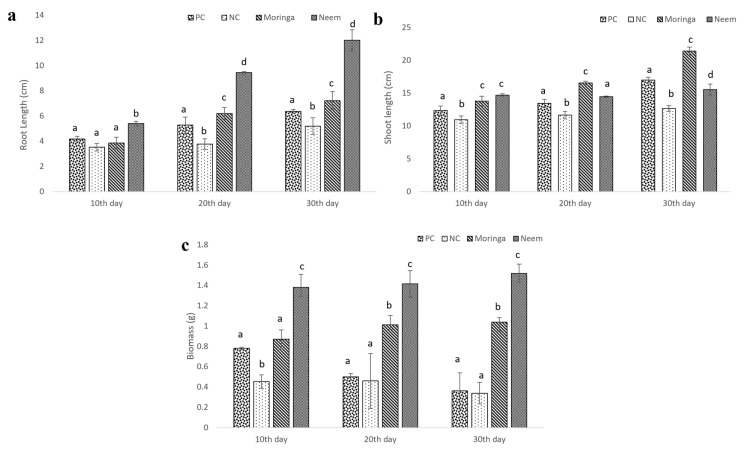
In vivo phyto stimulation of *A. esculentus* exposed to MO and AI gum polysaccharide. (**a**) Root growth, (**b**) shoot growth, (**c**) biomass. All tests are compared with positive and negative controls. Different letters on the bar graph denotes significant difference at *p* < 0.05.

Like the in vitro study, the effect of plant gum polysaccharide was measured using the parameters such as seed germination rate, root, shoot length, and biomass content that were observed every 10 days. The germination rate for seeds grown in both gum polysaccharide-enriched soils was found to be higher in comparison with both positive and negative control (Table 2). In *Moringa oleifera* gum treated, maximum growth was observed from the 10th day until 30th day for test plantlets. Likewise, the growth of plants was enhanced with *Azadirachta indica* gum polysaccharide. Poor-quality soil collected from the construction area (negative control) was showing reduced growth (Figure 10).

### 2.5. Soil Quality

**Figure 11:**
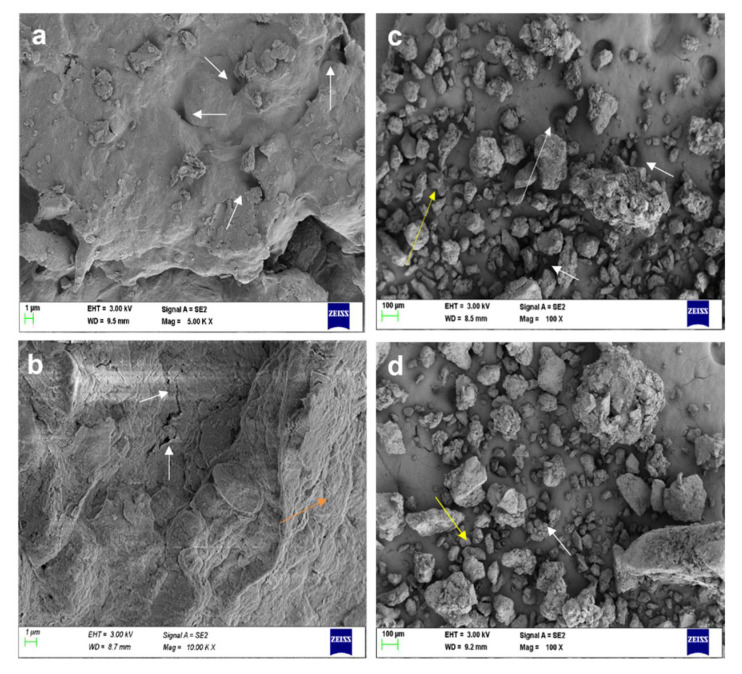

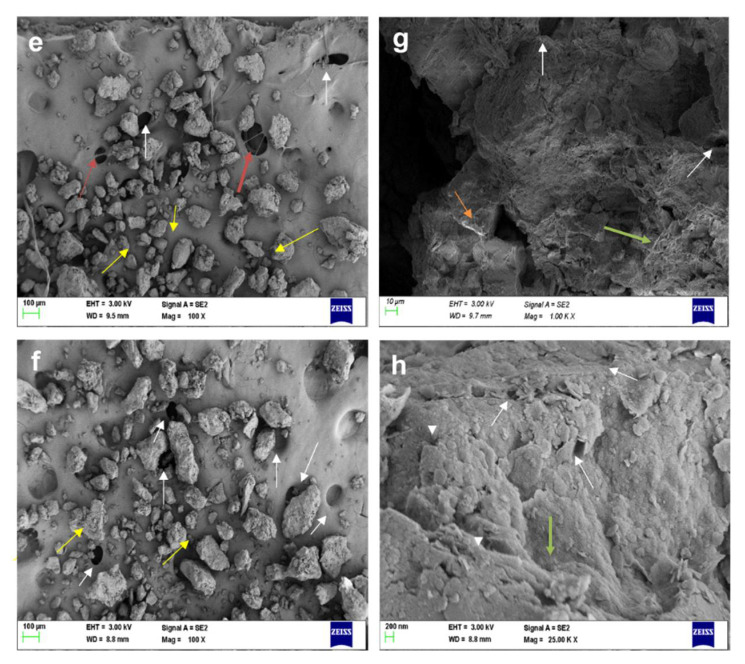
SEM analysis of soil. (**a**) Garden soil after 24 h, (**b**) garden soil after 30 days, (**c**) negative control soil after 24 h, (**d**) negative control soil after 30 days, (**e**) soil treated with MO gum after 24 h, (**f**) soil treated with MO gum after 30 days, (**g**) soil treated with AI gum after 24 h, (**h**) soil treated with AI gum after 30 days. White arrows denote the pores, green arrows denote the formation of clay mixture, and orange arrows denote biopolymer bridging, yellow arrows denote dispersed soil.

**Figure 12:**
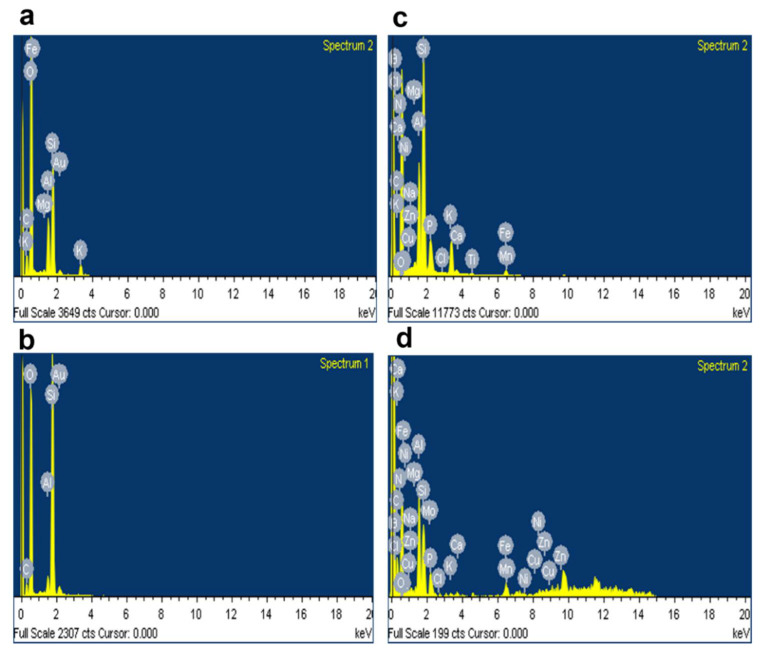

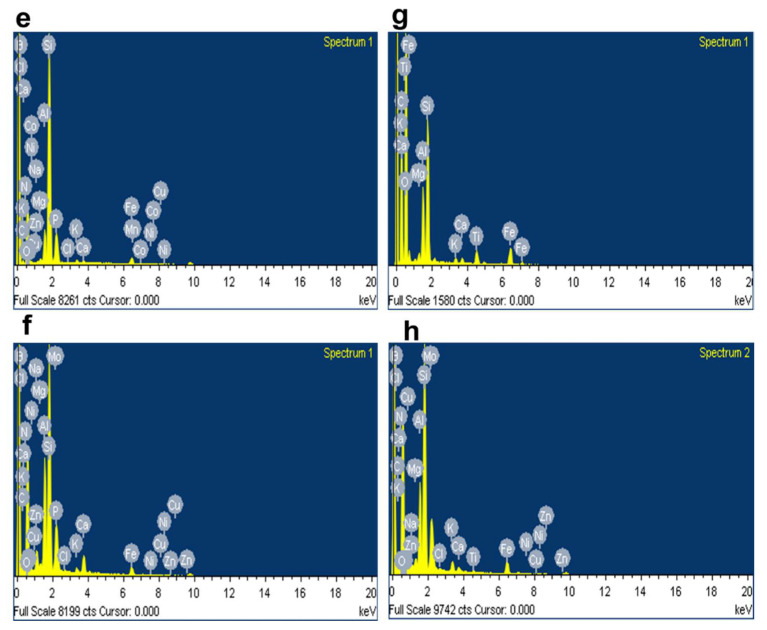
EDX analysis showing elemental composition of soil. (**a**) Garden soil after 24 h, (**b**) garden soil after 30 days, (**c**) negative control soil after 24 h, (**d**) negative control soil after 30 days, (**e**) soil treated with MO gum after 24 h, (**f**) soil treated with MO gum after 30 days, (**g**) soil treated with AI gum after 24 h, (**h**) soil treated with AI gum after 30 days.

The porosity of the soil was determined using the saturation method as mentioned in Matko [52], and the porosity for the MO gum treated soil was 37.5% and 35% in the case of AI gum treated soil (Table 3). Hence, it was evident from the experiment that the addition of gum improved the porosity of the soil than the negative control. Soil analysis was performed to analyze the macro- and micro-nutrients in the soil, and the results revealed the pH of the test soil to be 6.6 and 7.25 for MO and AI gum polysaccharide treated soil, respectively. Potassium and phosphorus components of the MO gum treated soil were found to be at 1.36 and 0.26%, whereas for the control soil, they were found to be 1.12 and 0.12%, respectively. Micronutrients do play an important role in the metabolic process [53,54]. Therefore, the addition of gum improved the quantity of these two nutrients, which could be the reason for the improved plant growth observed during the study. However, for AI gum treated soil, the potassium content was higher than the negative control soil, but the phosphorus in the gum polysaccharide-enriched soil was considerably lower than garden soil, which was reflected in the growth of the plants. Similar results were observed in another study where the improvements in these two nutrients were seen in the mixture of soil with enzymatically synthesized gum tragacanth-acrylic acid-based hydrogel, which was concluded to increase the soil fertility [55]. SEM analysis revealed the morphological changes that occurred in the soil after mixing with the gum polysaccharide. Stabilization of dispersive soil occurred due to the formation of biopolymer bonding between the void spaces, forming bridges between the dispersed sandy soil components after 24 h as well as to the 30th day (Figure 11e–h), and also increasing porosity, as evidenced by porosity analysis (Table 3). The images also revealed the presence of clay mixture, which could be the result of mixing the plant gum polysaccharide into the soil particles, which is said to increase the surface strength and stability of the particles (Figure 11). Similar results were observed in a study conducted by Ramachandran et al. [56]. The EDX analysis revealed the elemental composition present in the soil, which correlated with the soil analysis report (Figure 12). Previously, *Mangifera indica* gum has been reported to improve seed germination, vegetative growth, and seedling growth in *Capsicum frutescens*. It was also said to improve soil porosity and water retention capacity [3].

### 2.6. Pesticidal Activity

**Figure 13:**
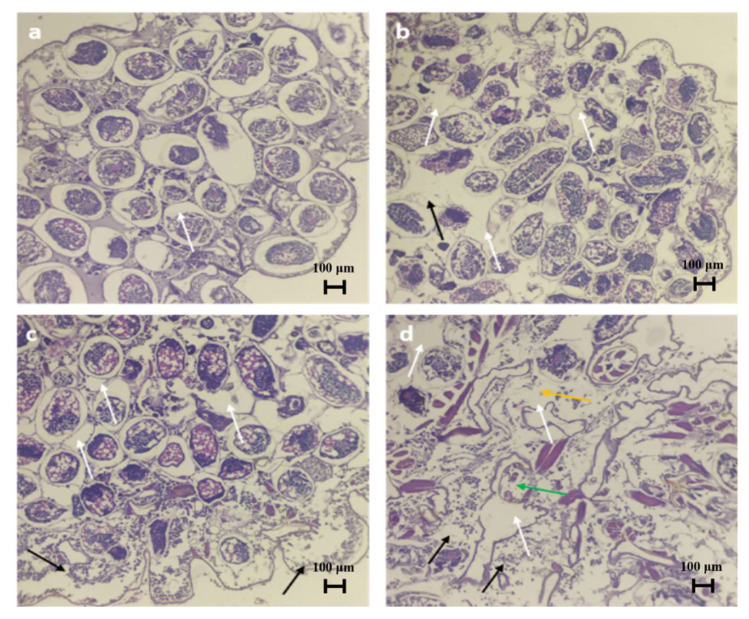
Histology of mealy bugs exposed to MO gum polysaccharide. (**a**) Control, (**b**) exposed to 10% MO, (**c**) exposed to 20% MO, (**d**) exposed to 30% MO. White arrows indicate interstitial spaces, black arrows indicate myolysis, orange arrow indicates total erosion of gut region, green arrow indicates degradation of gamma proteobacterial cells.

**Figure 14:**
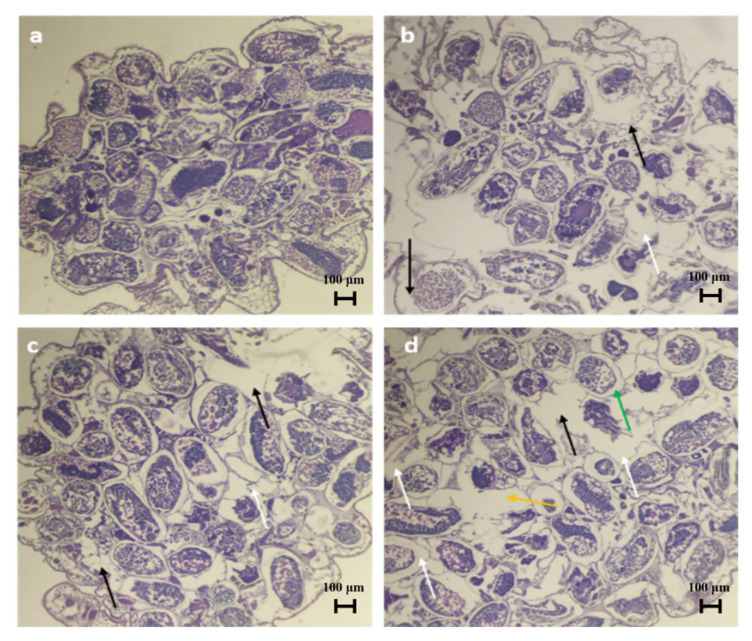
Histology of mealy bugs exposed to AI gum polysaccharide. (**a**) Control, (**b**) exposed to 10% AI, (**c**) exposed to 20% AI, (**d**) exposed to 30% AI. White arrows indicate interstitial spaces, black arrows indicate myolysis, orange arrow indicates total erosion of gut region, and green arrow indicates degradation of gamma proteobacterial cells.

Bio-pesticidal effects of plant gum polysaccharides were explored against mealy bugs through the mortality rate and histopathological images. The mortality rate was calculated at 24 h and 48 h of the study, and it was observed that the death rate was found to be high at a higher concentration and with increased exposure time in the case of both plant gum polysaccharides (Table 4). Histology images revealed the damage inflicted on the tissues of the mealy bugs. Degradation of the intestinal region was observed along with an increase in interstitial spaces. Erosion of gammaproteobacterial cells and muscles (myolysis) was observed, and at maximum concentration, the gut region was completely degraded (Figure 13 and Figure 14). The extent of damage in the case of both plant gum polysaccharides was found to be higher at increased concentrations. Therefore, the above-mentioned results prove that the plant gum polysaccharides can be effectively used as pesticides in agricultural fields. Guar gum-based film showed bioactivity against brown plant hopper, and the mortality rate was in the range between 46.67% and 82.22% [57]. Phytochemical substances found in plant products can be employed as larvicides, insect growth regulators, repellents, and ovipositional attractants [58]. Many parts of the *Azadirachta indica* tree have been reported to have pesticidal activity, with azadirachtin and salannin being the major components with insecticidal properties [59]. The *Moringa oleifera* tree has also been reported to have bio-pesticidal activity [60].

## 3. Discussion

In this study, two plant gum polysaccharides—from *Moringa oleifera* and *Azadirachta indica*—were scraped off from the barks of the trees, dried, powdered and used for further processing. The powdered gum polysaccharide was characterized using various methods. The peaks around 279 (MO) and 258 nm (AI) in UV-Vis spectroscopy may indicate the presence of glucose and the absorbance peaks around 210 nm confirm the presence of xylose [46], as previously reported. In a study, Samrot et al. [15] performed UV-Vis analysis of *Azadirachta indica* gum polysaccharide and suggested the presence of glucose and xylose with absorbance peaks at around 265 nm and at 210 nm, respectively. According to Udo et al. [61], the absorption maxima for gums from *Acacia senegal* and *Anacardium occidentale* trees were 210 and 200 nm, respectively. The basic polysaccharide structure was revealed by the intense peak in the range 1010 to 1030 cm^−1^ in FTIR spectra, indicating the stretching vibrations of the glycoside bridge (C–O–C) and confirming the presence of polysaccharides [62,63]. Thin-layer chromatography is a preliminary and highly convenient approach for identifying sugars with high sensitivity where we monosaccharides like glucose, rhamnose etc. The presence of carbohydrates and reducing sugars in *Araucaria heterophylla* gum reported by Gayathri and Sundara, [64] reinforces the preceding statement. Couso et al. [65] revealed the presence of glucose, mannose, glucuronic acid and rhamnose in a molar ratio of 4:1:1:1 in exopolysaccharide isolated from a Gram-negative bacterium, *Acetobacter xylinum*. Kumar et al. [66] also reported the presence of fructose and mannose in *Vina mungo*. Kharbade and Joshi, [67] revealed that the plant gums have small amounts of sugars such as mannose, glucose, arabinose, galactose, rhamnose, xylose, fucose, glucuronic acid and galacturonic acid linked with each other in various ways. Thin-layer chromatography revealed the presence of glucose, likewise Kassim [68] also revealed the presence of glucose and mannose in xanthan gum of bacterium *Xanthomonas campestris* using TLC.

Biostimulants are a type of chemical that is now used to aid in the growth and development of horticultural plants. The addition of polysaccharides in the form of biopolymers is known to improve seed germination and seedling growth [69]. Plant gums, include a mixture of calcium, magnesium, and potassium ions as well as a significant amount of sugar and protein, can contribute to the higher growth in plants. Ciereszko, [70] suggested that the presence of glucose and sucrose in the gum polysaccharide increases the growth and metabolic processes in plants. In another study, the addition of *Acacia senegal* gum and its nitrogenous derivatives was found to significantly improve the growth, which was comparable with the results observed in this study [35]. It is said that sugars and light play important roles in rose bud development by upregulating the expression and activity of vacuolar invertases, which increases sugar demand [71,72]. When employed in micropropagation as an alternative to agar, xanthan gum showed a favorable effect on the regenerative potential of some plants, which implies its bio stimulative function [73]. Polysaccharide chains of carrageenan can boost root and shoot development, as well as improve net photosynthesis and basal and secondary metabolism in plants [74]. In a study by Salachna and Pietrak, [75], a biostimulant (carrageenan-depolymerized chitosan and xanthan-depolymerized chitosan) coating on the bulbs of *Eucomis autumnalis* proved to be a promising and ecologically friendly approach to boost plant growth that can be suggested for long-term ornamental plant production.

A stable and continuous pore system is required to form a well-defined soil structure that is essential for favorable plant growth. It is also one of the most useful parameters to determine whether the soil system can be characterized as a plant medium for plant growth [76]. A well-aerated porous soil is said to promote better plant growth, as it can increase the rate at which water and nutrients permeate the soil [77]. The increased swelling rates of AI and MO gums can contribute to higher moisture-retaining capacity. In the realm of agriculture, the use of gum-based hydrogels consisting of xanthan, cassava, and chitosan has been reported to prolong moisture availability due to improved water retention capacity in the form of a matrix for the controlled release of fertilizers, nutrients, and pesticides in soil [78]. The addition of polymers such as xanthan and cellulose to the soil reduced the rate of soil erosion by 80% [79]. Microscopic investigation of sand mixed with xanthan gum polysaccharide revealed biopolymer bridging between pores and the formation of biopolymer clay matrix [56]. Likewise, in another study, strong bonding between the void gaps of dispersive soils were observed after the addition of xanthan gum [80]. Plant gum-based stimulants can be prepared and supplied to plants to increase the fertility of the soil as well as to enhance the growth of slow-growing plants.

Mealybugs (*Phenacoccus solenopsis*) are covered with vast amounts of white waxy material and can be found on shoot tips and the undersides of leaves. They are usually oval in shape and clump together to form a bunch. They also deform the stems, dry up the leaves and infest all parts of the host plants except the roots. Different parts of MO and AI trees have been reported to have pesticidal activities [81,82]. As expected, AI and MO gums also showed a high degree of dose-dependent pesticidal activity against mealy bugs. Utilization of plant-based biopesticides can be environmentally friendly and safe to the non-target organisms.

## 4. Materials and Methods

### 4.1. Collection and Powdering of Gum

All methods were performed in accordance with the relevant guidelines and regulations. Leaves of *Moringa oleifera* and *Azadirachta indica* were collected and authenticated by Dr. P Jayaraman, Director, Plant Anatomy Research Centre, Chennai with authentication numbers PARC/2021/4475 and PARC/2021/4473, respectively. The gum exudates of *Moringa oleifera* (MO) and *Azadirachta indica* (AI) were scraped off from the barks of trees found in our garden in Chennai, Tamil Nadu. The obtained gums were subjected to complete drying and later powdered and stored in an airtight container. The powdered gums were used for further study and characterization (Supplementary Figures S1 and S2).

### 4.2. Purification and Characterization

Qualitative phytochemical analyses for the presence of alkaloids, flavonoids, saponins, reducing sugars, amino acids, phytosterols, tannins, and glycosides were performed for the crude gums (MO and AI) [83,84,85,86]. Alkaline reagent test for flavonoids, froth test for saponins, Benedict’s test for reducing sugars, Ninhydrin’s test for amino acids, Salkowski reaction test for phytosterols, and potassium dichromate test for tannins were performed. The crude gums were also subjected to UV-Visible spectrophotometric analysis (Shimadzu UV-1800, Kyoto, Japan) in the spectral range from 200 to 800 nm. Fourier transform infrared spectroscopy (Shimadzu, IRTracer 100, Kyoto, Japan) was performed to identify the functional groups present in the samples. TLC was performed on a TLC silica plate (Merck, F_245_) for the MO and AI gums using the solvent systems ethanol:water (2:1) and ethanol:chloroform (2:1), respectively. Bands were visualized by exposing the samples to an iodine-saturated chamber and also by spraying DPPH (on the plates which were not exposed to iodine). The MO and AI gum polysaccharides were subjected to phenol sulfuric acid assay to identify the presence of monosaccharides [5,87]. Ash and moisture contents in the MO and AI gums were determined using methods followed by Moreno et al. [88] and Gundidza et al. [89].

Gum polysaccharide (MO and AI) was purified by following the method of Samrot et al. [5] and was further derivatized to determine the composition of monosaccharides present in the plant gums using GC-MS analysis (Shimadzu, QP2010 PLUS, Kyoto, Japan). The procedure was performed as follows: 1 g of purified sample was added to 2 mL of 2 M trifluoroacetic acid (TFA) under heat for 6 h. The solution was further dissolved in 10 mg of hydroxylamine hydrochloride and 0.5 mL of pyridine at 90 °C for 30 min. Thus, acetylation was achieved. The mixture was cooled at room temperature and 0.5 mL of acetic anhydride was added, followed by vigorous mixing. The mixture was again placed in a hot water bath for 30 min and then cooled to room temperature. One microliter of the supernatant was subjected to GC-MS analysis to identify the monosaccharides presence in the gum samples [90,91]. The injection temperature was set at 240 °C and the detector was set such that the column temperature was increased to 240 °C at 5 °C/min; nitrogen gas was used as carrier. 

The purified polysaccharide was dissolved in distilled water and characterized using UV-Vis analysis (Shimadzu UV-1800, Japan) and FTIR analysis (Shimadzu, IRTRACER 100, Japan). TGA was conducted on the polysaccharides to determine their thermal degradative properties (SDT Q600 V20.9 Build 20). ^1^H and ^13^C nuclear magnetic resonance (NMR) spectra were also recorded using 400 MHz NMR (Bruker, Rheinstetten, Germany).

### 4.3. In-Vitro Study of Plant Growth

#### 4.3.1. Pre-Treatment of Seeds

*Abelmoschus esculentus* seeds were obtained from a local vendor and utilized for this study, as they are easy to grow, and the seed size meets the primary requirement for any seed handling system [92]. The seeds were surface sterilized with 2% sodium hypochlorite for 2 min and then soaked in distilled water overnight. Then, the soaked seeds were again rinsed with sodium hypochlorite solution to prevent any fungal growth during the study [93].

#### 4.3.2. Treatment of Seeds with Plant Gum Polysaccharides

An in-vitro study of the effect of gum polysaccharides on plant growth was carried out for a period of 9 days. One milliliter of a 10% plant gum polysaccharide (MO and AI) solution was applied to the seeds, which were placed in a cotton bed in clean, air-dried petri dishes. The setup was maintained in a humid chamber at room temperature, and the seeds were sprayed with distilled water at every 24 h to maintain moisture content. The seed germination and growth with the study concentrations were observed on every 3rd consecutive day [94].

$$\mathrm{Germination}\;\mathrm{rate}\;=\frac{\mathrm{Total}\;\mathrm{number}\;\mathrm{of}\;\mathrm{seeds}\;\mathrm{germinated}}{\mathrm{Total}\;\mathrm{number}\;\mathrm{of}\;\mathrm{seed}\;\mathrm{used}}\;$$

#### 4.3.3. Growth and Biomass Measurement

The root and shoot growth along with biomass were observed on every 3rd day for the period of 9 days. The total weight of each plant was weighed to calculate the biomass. The observations for the study were performed in triplicates.

### 4.4. In-Vivo Study of Effect of Gum Polysaccharide on Plant Growth and Soil

#### 4.4.1. Treatment of Soil with Plant Gum Polysaccharide

The effect of plant gum on improvement of soil quality was studied in-vivo for a period of 30 days. Poor-quality soil was collected from a construction site in Tambaram, Chennai, Tamil Nadu. A 10% plant gum solution was mixed thoroughly with a pot containing poor-quality soil for uniform dispersion. The mixture was left undisturbed for 24 h in a closed container, after which the seeds were sown in the soil. Seed germination and growth were observed on every 10th consecutive day. Poor-quality soil without plant gum was used as negative control, and garden soil enriched with cow dung manure was used as positive control.

#### 4.4.2. Growth and Biomass Measurement

The root and shoot growth along with biomass were measured on every 10th day for a period of 30 days. The total weight of the plant was weighed to calculate the biomass. The observations for the study were performed in triplicates.

#### 4.4.3. Soil Quality

To test the effect of gum polysaccharide on soil, the following parameters were observed: soil porosity [52], water holding capacity [95], quantification of micro and macro nutrients and SEM-EDX analysis (Zeiss Ultra Plus, Oberkochen, Germany). All the results were compared with the negative (collected from construction site) and positive control (garden) soil.

### 4.5. Biopesticidal Activity

The plant gum polysaccharides obtained from *Moringa oleifera* and *Azadirachta indica* were also investigated for their pesticidal activity. The study was conducted for 48 h under room temperature. Mealybugs collected from *Hibiscus* sp. infested plants were utilized for this study. The mealybugs were exposed to different concentrations of both plant gum polysaccharides—MO and AI (10%, 20% and 30%). Four clean, air-dried petri dishes were used for each plant gum polysaccharide, which was applied onto the surfaces of the dishes. Ten mealy bugs per plate were released onto the surface of the plant gum polysaccharide-treated petri dishes, and host leaves were also added as feed for the organisms. The open surface of the petri dish was covered with moist filter paper (Whatman filter paper) and holes were punched into the papers for aeration [96]. The mortality rate of the mealybugs was calculated at every 24 h (distilled water was used as control). Histopathological studies were performed on the mealybugs after 48 h to observe the damage inflicted on their tissues.

### 4.6. Statistical Analysis

All experiments were carried out in triplicates and represented as mean ± standard deviation. Test results were compared with control after performing one-way ANOVA with Tukey HSD.

## 5. Conclusions

In this study, two plant gum polysaccharides—from *Moringa oleifera* and *Azadirachta indica*—were scraped off from the barks of the trees, dried, powdered and used for further processing. Characterization of the gum polysaccharides revealed the presence of various monosaccharides including glucose, galactose, xylose etc., which are believed to enhance the plant growth. Seeds exposed to gums were found to increase the root and shoot development and also improved the soil porosity and quality. Furthermore, histology studies revealed that the plant gum could cause severe impact in gut of mealybug, thus can be used as biopesticides.

## Figures and Tables

**Table 1:** Germination rate.

Control	*Moringa oleifera*	*Azadirachta indica*
75% ^a^	85% ^b^	60% ^c^

Different letters on the values denote significant difference at *p* < 0.05.

**Table 2:** Germination rate.

Positive Control	Negative Control	MO Gum Polysaccharide	AI Gum Polysaccharide
95% ^a^	85% ^b^	100% ^c^	100% ^c^

Different letters on the values denote significant difference at *p* < 0.05.

**Table 3:** Soil analysis of gum polysaccharide-treated soils.

S. No	Parameter (Units)	Garden Soil	Negative Control Soil	*Moringa oleifera* Enriched Soil	*Azadirachta indica* Enriched Soil
1	pH	8.0 ± 0.1643 ^a^	6.81 ± 0.1502 ^b^	6.66 ± 0.1015 ^b^	7.25 ± 0.1936 ^c^
2	Porosity (%)	49 ± 0.8649 ^a^	27.5 ± 0.6221 ^b^	37.5 ± 0.9555 ^c^	35 ± 0.8289 ^c^
3	Water holding capacity (%)	80 ± 0.994 ^a^	40 ± 0.9471 ^b^	60 ± 0.7906 ^c^	70 ± 0.7537 ^d^
4	Conductivity (µs/cm)	138 ± 0.7537 ^a^	158.4 ± 0.7232 ^b^	124.3 ± 0.719 ^c^	125.6 ± 1.0756 ^c^
5	Moisture (%)	17 ± 0.5357 ^a^	15.27 ± 0.5062 ^b^	16.01 ± 0.6736 ^c^	15.2 ± 0.5595 ^b^
6	Available phosphorus as P (%)	186.68 ± 0.6981 ^a^	0.12 ± 0.0071 ^b^	0.26 ± 0.0164 ^b^	6.93 ± 0.2853 ^c^
7	Potassium as K (%)	351.6 ± 0.3847 ^a^	1.12 ± 0.0989 ^b^	1.36 ± 0.1182 ^b^	2.8 ± 0.2762 ^c^
8	Total organic carbon (%)	1.01 ± 0.0903 ^a^	0.95 ± 0.0305 ^b^	0.92 ± 0.0342 ^b^	0.65 ± 0.0321 ^c^
9	Na (mg/100 g)	214 ± 3.9115 ^a^	542.3± 6.5221 ^b^	621.3 ± 4.7379 ^c^	559 ± 12.6044 ^b^
10	Soluble Ca (g/100 g)	347 ± 5.6125 ^a^	26.22 ± 2.1083 ^b^	14.90 ± 0.6427 ^c^	28.6 ± 9.9863 ^b^
11	Soluble Mg (g/100 g)	229 ± 9.8742 ^a^	7.36 ± 2.0748 ^b^	4.52 ± 1.1333 ^b^	39.31 ± 10.1419 ^c^
12	SO_4_ (mg/kg)	BQL (LoQ: 0.001)	9.72 ± 2.0415 ^a^	8.15 ± 1.7437 ^b^	8 ± 4.2435 ^b^
13	Cl (mg/100 g)	BQL (LoQ: 0.001)	0.79 ± 0.1132 ^a^	0. 321 ± 0.0216 ^b^	0.4 ± 0.1029 ^b^
14	N (g/100 g)	28.4 ± 4.396 ^a^	0.18 ± 0.0114 ^b^	0.16 ± 0.013 ^b^	0.27 ± 0.013 ^b^
15	Fe (mg/kg)	25.07 ± 1.3375 ^a^	14.3 ± 1.8321 ^b^	14.20 ± 3.5829 ^b^	12.08 ± 3.9558 ^c^
16	Cu (mg/kg)	5.51 ± 0.0158 ^a^	BQL (LoQ: 0.001)	BQL (LoQ: 0.001)	BQL (LoQ: 0.001)
17	Mn (mg/kg)	3.62 ± 0.0418 ^a^	BQL (LoQ: 0.001)	BQL (LoQ: 0.001)	BQL (LoQ: 0.001)
18	Zn (mg/kg)	7.28 ± 1.8231 ^a^	16.12 ± 3.8269 ^b^	14.20 ± 3.8932 ^c^	11.3 ± 4.3522 ^d^
19	Mo (mg/kg)	BQL (LoQ: 0.001)	BQL (LoQ: 0.001)	BQL (LoQ: 0.001)	BQL (LoQ: 0.001)
20	B (mg/kg)	2.1 ± 0.0412 ^a^	BQL (LoQ: 0.001)	BQL (LoQ: 0.001)	BQL (LoQ: 0.001)
21	Ni (mg/kg)	BQL (LoQ: 0.001)	BQL (LoQ: 0.001)	BQL (LoQ: 0.001)	BQL (LoQ: 0.001)

BQL: Below quantifiable limit, LoQ: Limit of quantitation. Different letters on the values denote significant difference at *p* < 0.05.

**Table 4:** Mortality rate with plant gum polysaccharide.

Concentration	MO	AI	MO	AI	MO	AI
	10%	10%	20%	20%	30%	30%
24 h	10%	-	30%	20%	30%	40%
48 h	40%	40%	50%	50%	60%	50%

## Data Availability

Not applicable.

## Supplementary Materials

The following supporting information can be downloaded at: https://www.mdpi.com/article/10.3390/molecules27123720/s1, Figure S1. *Moringa oleifera* gum (MO), (A) Dried crude gum, (B) Powdered gum; Figure S2. *Azadirachta indica* gum (AI), (A) Dried crude gum, (B) Powdered gum; Table S1. Phytochemical analysis of gum polysaccharides; Figure S3. UV-Vis analysis (a) MO gum, (b) AI gum; Figure S4. FTIR spectrum (a) MO gum, (b) AI gum; Figure S5. TLC of gum polysaccharide of MO gum (a) exposed to Iodine, (b) sprayed using DPPH; Figure S6. TLC of gum polysaccharide AI gum (a) exposed to Iodine, (b) sprayed using DPPH; Figure S7. Phenol Sulfuric assay (a) MO gum, (b) AI gum; Figure S8. UV-Vis analysis purified gum polysaccharide (a) MO gum, (b) AI gum; Figure S9. FTIR analysis purified gum polysaccharide (a) MO gum, (b) AI gum.

## Sample Availability

Samples of the compounds are not available from the authors.
