# Supplementary material for: Purification and Characterization of Gum-Derived Polysaccharides of Moringa oleifera and Azadirachta indica and Their Applications as Plant Stimulants and Bio-Pesticidal Agents
Source: Molecules. 2022 Jun 9;27(12):3720. doi: 10.3390/molecules27123720 (PMC9230390; doi:10.3390/molecules27123720)
Supplement: Supplementary file 1 [file molecules-27-03720-s001.zip › molecules-1569186-supplementary.pdf]

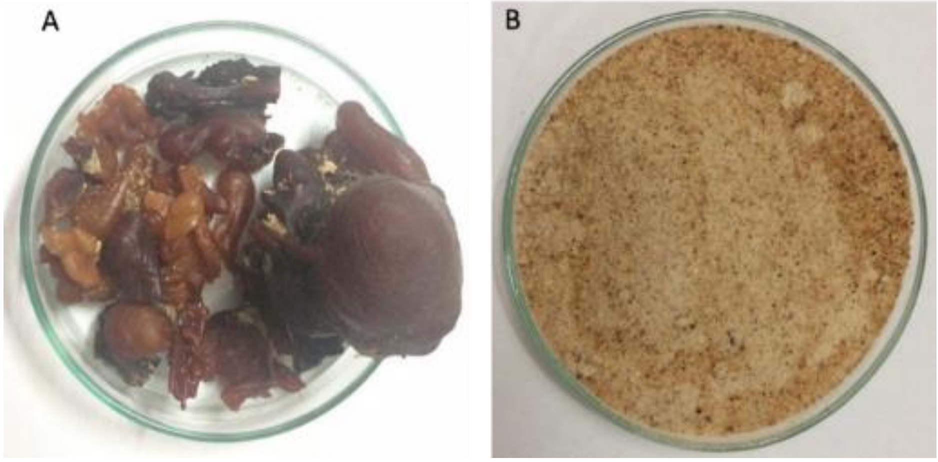

Figure S1. *Moringa oleifera* gum (MO), a) Dried crude gum, b) Powdered gum.

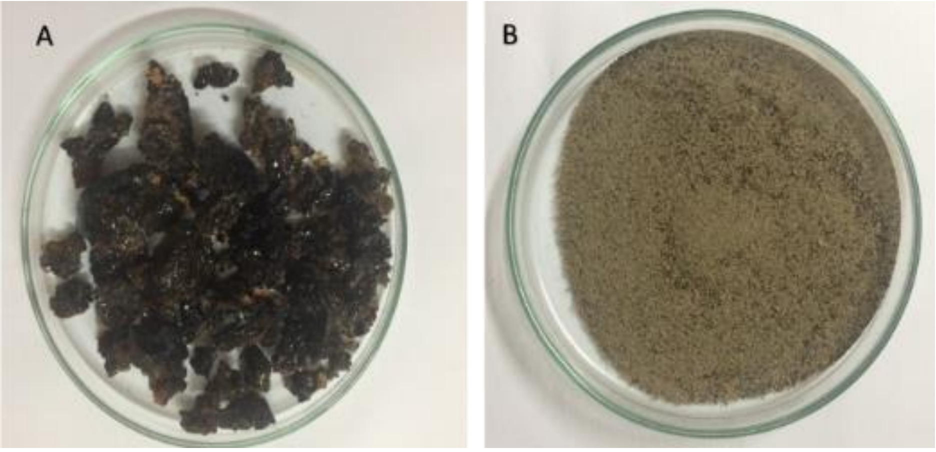

Figure S2. *Azadirachta indica* gum (AI), a) Dried crude gum, b) Powdered gum.

Test	MO	AI
Alkaloids	++	+
Flavonoids	-	++
Saponins	+	+
Reducing Sugar	+	+
Amino acids	-	-

Phytosterols	+	-
Tannins	+	+
Glycosides	+	+

Table S1. Phytochemical analysis of gum.

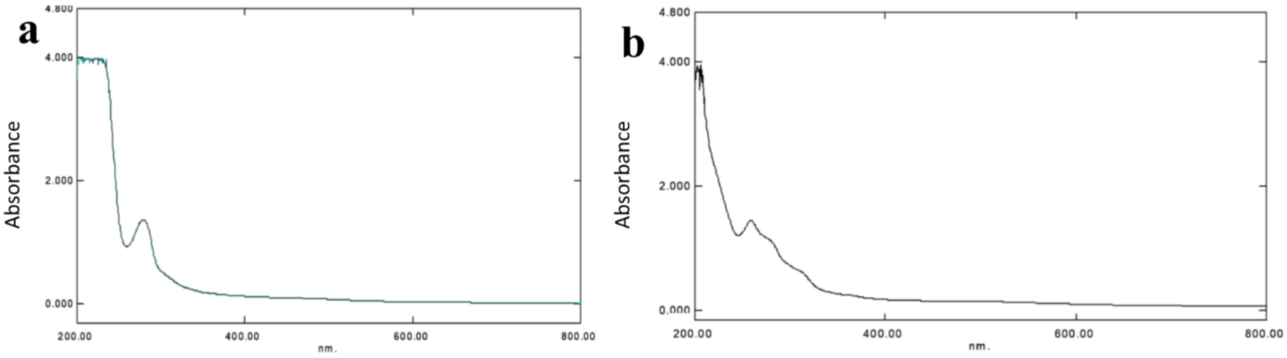

Figure S3. UV-Vis analysis a) MO gum, b) AI gum.

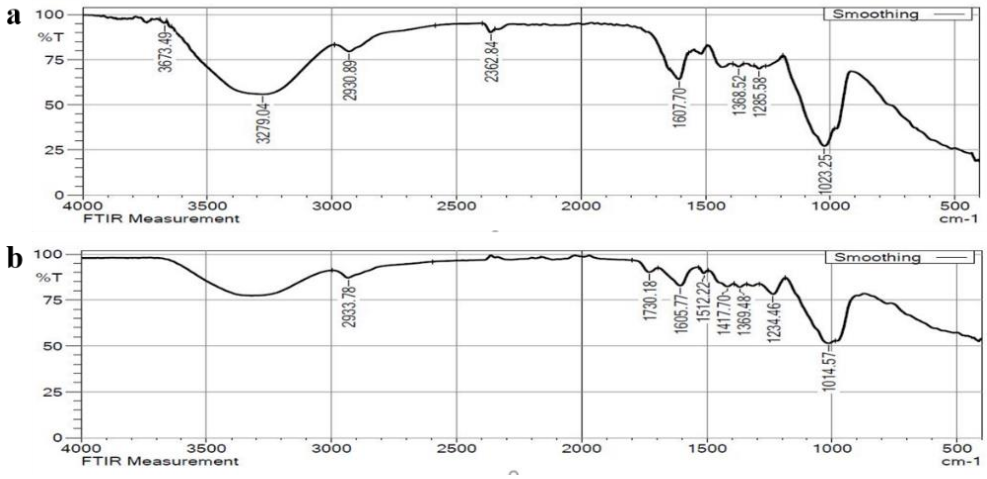

Figure S4. FTIR spectrum a) MO gum, b) AI gum.

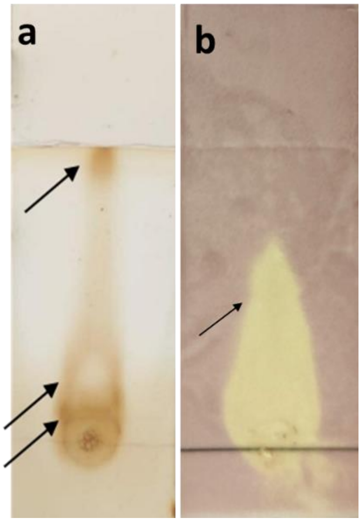

Figure S5. TLC of gum polysaccharide of MO gum a) exposed to Iodine, b) DPPH sprayed.

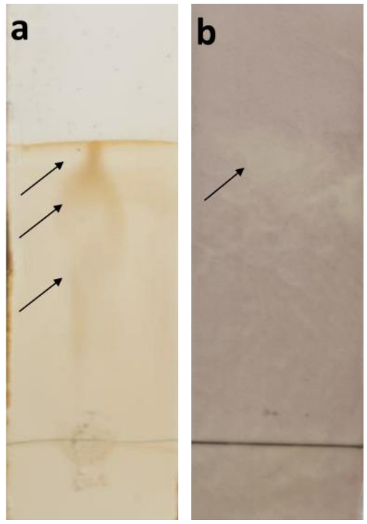

Figure S6. TLC of gum polysaccharide AI gum a) exposed to Iodine, b) DPPH sprayed.

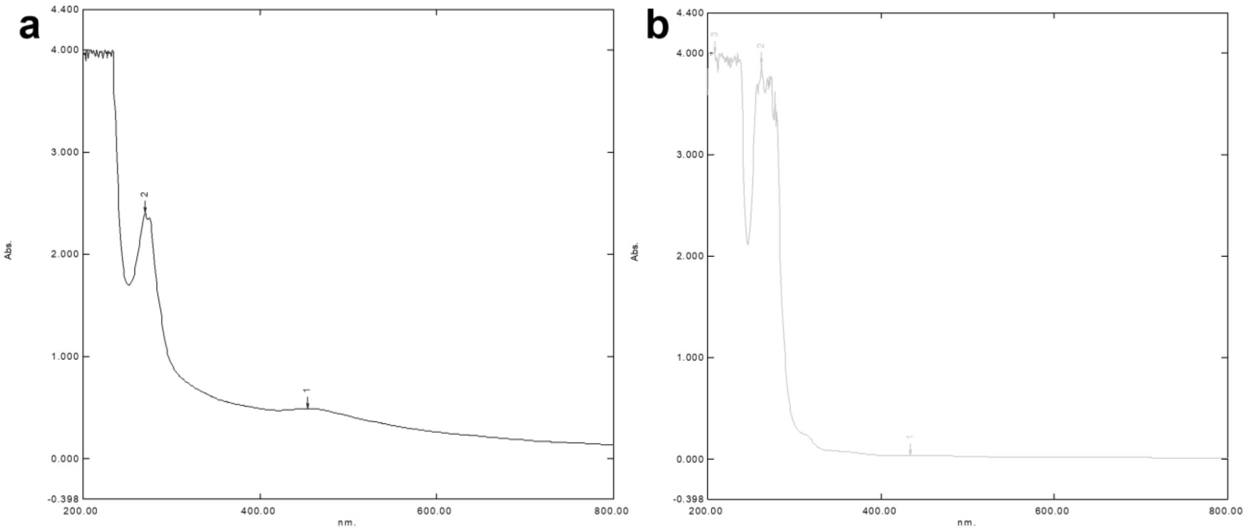

Figure S7. Phenol Sulfuric assay a) MO gum, b) AI gum.

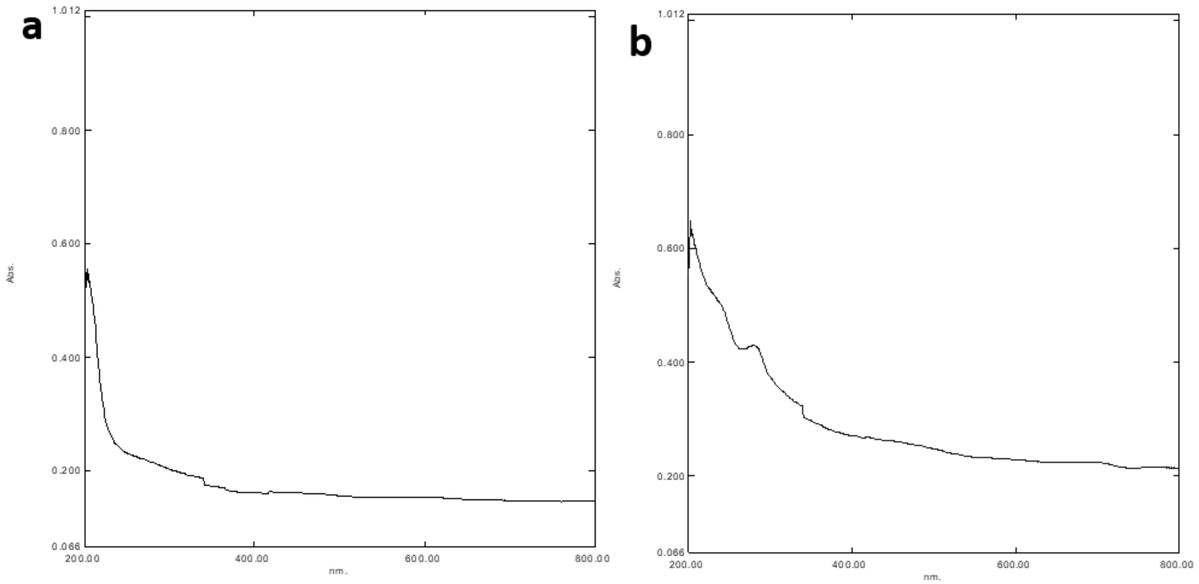

Figure S8. UV-Vis analysis purified gum polysaccharide a) MO gum, b) AI gum.

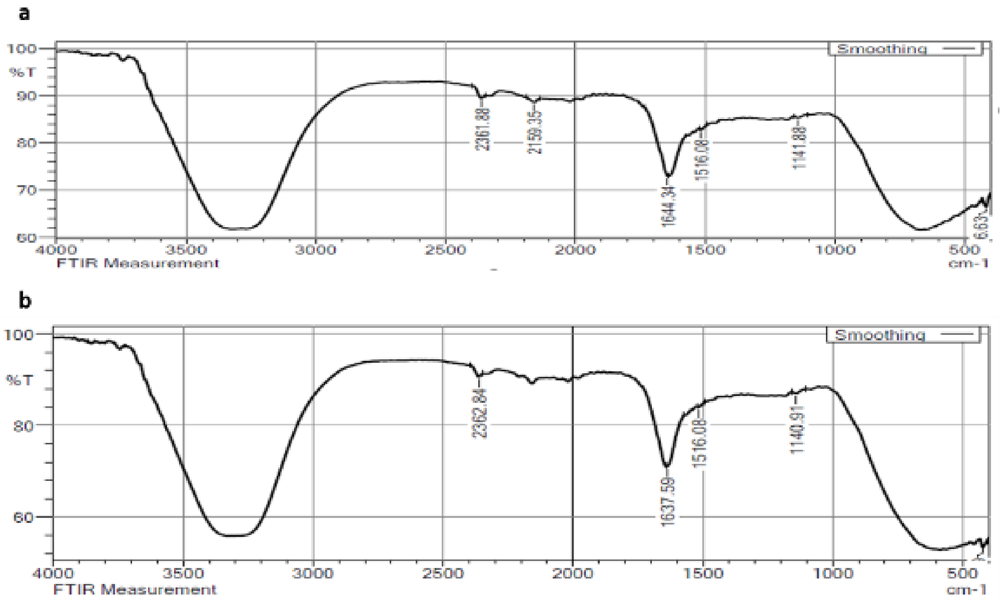

Figure S9. FTIR analysis purified gum polysaccharide a) MO gum, b) AI gum.
